# The complete genome sequence of *Clostridium indolis* DSM 755^T^

**DOI:** 10.4056/sigs.5281010

**Published:** 2014-03-18

**Authors:** Amy S. Biddle, Susan Leschine, Marcel Huntemann, James Han, Amy Chen, Nikos Kyrpides, Victor Markowitz, Krishna Palaniappan, Natalia Ivanova, Natalia Mikhailova, Galina Ovchinnikova, Andrew Schaumberg, Amrita Pati, Dimitrios Stamatis, Tatiparthi Reddy, Elizabeth Lobos, Lynne Goodwin, Henrik P. Nordberg, Michael N. Cantor, Susan X. Hua, Tanja Woyke, Jeffrey L. Blanchard

**Affiliations:** 1 Department of Microbiology, University of Massachusetts, Amherst, MA, USA; ^2^Institute for Cellular Engineering, University of Massachusetts, Amherst, MA, USA ^3^Department of Veterinary and Animal Sciences, University of Massachusetts, Amherst, MA, USA; 4 Joint Genome Institute, Walnut Creek, CA, USA; 5 Department of Biology, University of Massachusetts, Amherst, MA, USA; 6 Graduate Program in Organismal and Evolutionary Biology, University of Massachusetts, Amherst, MA, USA.

**Keywords:** *Clostridium indolis*, citrate, lactate, aromatic degradation, nitrogen fixation, bacterial microcompartments

## Abstract

*Clostridium indolis* DSM 755^T^ is a bacterium commonly found in soils and the feces of birds and mammals. Despite its prevalence, little is known about the ecology or physiology of this species. However, close relatives, *C. saccharolyticum* and *C. hathewayi*, have demonstrated interesting metabolic potentials related to plant degradation and human health. The genome of *C. indolis* DSM 755^T^ reveals an abundance of genes in functional groups associated with the transport and utilization of carbohydrates, as well as citrate, lactate, and aromatics. Ecologically relevant gene clusters related to nitrogen fixation and a unique type of bacterial microcompartment, the CoAT BMC, are also detected. Our genome analysis suggests hypotheses to be tested in future culture based work to better understand the physiology of this poorly described species.

##  Introduction

**Figure 1:**
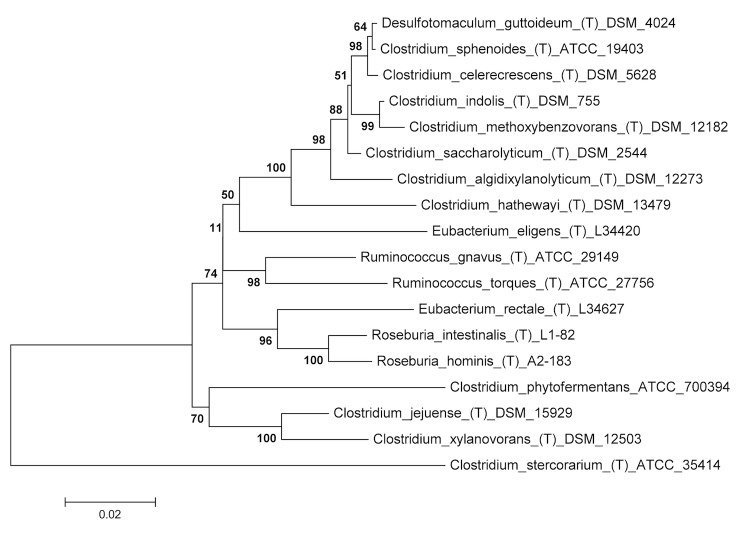
Phylogenetic tree based on 16S rRNA gene sequences highlighting the position of *Clostridium indolis* relative to other type strains (T) within the *Lachnospiraceae*. The strains and their corresponding NCBI accession numbers (and, when applicable, draft sequence coordinates) for 16S rRNA genes are: *Desulfotomaculum guttoideum* strain DSM 4024^T^, Y11568; *C. sphenoides* ATCC 19403^T^, AB075772; *C. celerecrescens* DSM 5628^T^, X71848; *C. indolis* DSM 755^T^, Pending release by JGI: 1620643-1622056; *C. methoxybenzovorans* SR3, AF067965; *C. saccharolyticum* WM1^T^, NC_014376:18567-20085; *C. algidixylanolyticum* SPL73^T^, AF092549; *C. hathewayi* DSM 13479^T^, ADLN00000000: 202-1639; *Eubacterium eligens* L34420 ^T^, L34420; *Ruminococcus gnavus* ATCC 29149^T^, X94967; R. torques ATCC 27756^T^, L76604; *E. rectale* L34627^T^; *Roseburia intestinalis* L1-82^T^, AJ312385; *R. hominis* A2-183^T^, AJ270482; *C. jejuense* HY-35-12^T^, AY494606; *C. xylanovorans* HESP1^T^, AF116920; *C. phytofermentans* ISDg^T^, CP000885: 15754-17276. The tree uses sequences aligned by MUSCLE, and was inferred using the Neighbor-Joining method [2]. The optimal tree with the sum of branch lengths = 0.50791241 is shown. The percentage of replicate trees in which the associated taxa clustered together in the bootstrap test (500 replicates) are shown next to the branches [3]. The tree is drawn to scale, with branch lengths in the same units as those of the evolutionary distances used to infer the phylogenetic tree. The evolutionary distances were computed using the Maximum Composite Likelihood method [4] and are in the units of the number of base substitutions per site. Evolutionary analyses were conducted in MEGA 5 [5]. *C. stercorarium* ATCC 35414^T^, CP003992: 856992-858513 was used as an outgroup.

The *C. saccharolyticum* species group is a poorly described and taxonomically confusing clade in the *Lachnospiraceae*, a family within the *Clostridiales* that includes members of clostridial cluster XIVa [1]. This group includes *C. indolis*, *C. sphenoides*, *C. methoxybenzovorans*, *C. celerecrescens*, and *Desulfotomaculum guttoideum*, none of which are well studied (Figure 1). *C. saccharolyticum* has gained attention because its saccharolytic capacity was shown to be syntrophic with the cellulolytic activity of *Bacteroides cellulosolvens* in co-culture, enabling the conversion of cellulose to ethanol in a single step [6,7]. Members of this group, such as *C. celerecrescens,* are themselves cellulolytic [8], and others are known to degrade unusual substrates such as methylated aromatic compounds (*C. methoxybenzovorans*) [9], and the insecticide lindane (*C. sphenoides*) [10]. *C. indolis* was targeted for whole genome sequencing to provide insight into the genetic potential of this taxa that could then direct experimental efforts to understand its physiology and ecology.

## Classification and features

The general features of *Clostridium indolis* DSM 755^T^ are listed in Table 1. *C. indolis* DSM 755^T^ was originally named for its ability to hydrolyze tryptophan to indole, pyruvate, and ammonia [23] in the classic Indole Test used to distinguish bacterial species. It has been isolated from soil [24], feces [25], and clinical samples from infections [27]. Despite its prevalence, *C. indolis* is not well characterized, and there are conflicting reports about its physiology. It is described as a sulfate reducer with the ability to ferment some simple sugars, pectin, pectate, mannitol, and galacturonate, and convert pyruvate to acetate, formate, ethanol, and butyrate [28]. According to this source, neither lactate nor citrate are utilized, however other studies demonstrate that fecal isolates closely related to *C. indolis* may utilize lactate [29], and that the type strain DSM 755^T^ utilizes citrate [30]. It is unclear whether *C. indolis* is able to make use of a wider range of sugars or break down complex carbohydrates, however growth is reported to be stimulated by fermentable carbohydrates [28].

**Table 1:** Classification and general features of *Clostridium indolis* DSM 755^T^

**MIGS ID**	**Property**	**Term**	**Evidence Code**
		Domain *Bacteria*	TAS [11]
		Phylum *Firmicutes*	TAS [12-14]
		Class *Clostridia*	TAS [15,16]
	Current classification	Order *Clostridiales*	TAS [17,18]
		Family *Lachnospiraceae*	TAS [15,19]
		Genus *Clostridium*	TAS [17,20,21]
		Species *Clostridium indolis*	TAS [17,22]
		Type strain DSM 755	
	Gram stain	Negative	TAS [23,24]
	Cell shape	Rod	TAS [23,24]
	Motility	Motile	TAS [23,24]
	Sporulation	Terminal, spherical spores	TAS [23,24]
	Temperature range	Mesophilic	TAS [23,24]
	Optimum temperature	37^o^C	TAS [23,24]
	Carbon sources	Glucose, lactose, sucrose, mannitol, pectin, pyruvate, others	TAS [23,24]
	Terminal electron receptor	Sulfate	TAS [23,24]
	Indole test	Positive	TAS [23,24]
MIGS-6	Habitat	Isolated from soil, feces, wounds	TAS [24,25]
MIGS-6.3	Salinity	Inhibited by 6.5% NaCl	TAS [23,24]
MIGS-22	Oxygen	Anaerobic	TAS [23,24]
MIGS-15	Biotic relationship	Free living and host associated TAS [24,25],9	
MIGS-14	Pathogenicity	No NAS	
MIGS-4	Geographic location	Soil, feces TAS [24,25],9	

Evidence codes - IDA: Inferred from Direct Assay; TAS: Traceable Author Statement (i.e., a direct report exists in the literature); NAS: Non-traceable Author Statement (i.e., not directly observed for the living, isolated sample, but based on a generally accepted property for the species, or anecdotal evidence). These evidence codes are from the Gene Ontology project [26].

## Genome sequencing information

### Genome project history

The genome was selected based on the relatedness of *C. indolis* DSM 755^T^ to *C. saccharolyticum*, an organism with interesting saccharolytic and syntrophic properties. The genome sequence was completed on May 2, 2013, and presented for public access on June 3, 2013. Quality assurance and annotation done by DOE Joint Genome Institute (JGI) as described below. Table 2 presents a summary of the project information and its association with MIGS version 2.0 compliance [31].

**Table 2:** Project information

**MIGS ID**	**Property**	**Term**
MIGS-31	Finishing quality	Improved Draft
MIGS-28	Libraries used	Shotgun and long insert mate pair (Illumina), SMRTbell^TM^ (PacBio)
MIGS-29	Sequencing platforms	Illumina and PacBio
MIGS-31.2	Fold coverage	759.7× (Illumina), 51.6× (PacBio)
MIGS-30	Assemblers	Velvet, AllpathsLG
MIGS-32	Gene calling method	Prodigal, GenePRIMP
	Genome Database release	June 3, 2013 (IMB)
	Genbank ID	Pending release by JGI
	Genbank Date of Release	Pending release by JGI
	GOLD ID	Gi22434
	Project relevance	Anaerobic plant degradation

### Growth conditions and DNA isolation

*C. indolis* DSM 755^T^ was cultivated anaerobically on GS2 medium as described elsewhere [32]. DNA for sequencing was extracted using the DNA Isolation Bacterial Protocol available through the JGI (http://www.jgi.doe.gov). The quality of DNA extracted was assessed by gel electrophoresis and NanoDrop (ThermoScientific, Wilmington, DE) according to the JGI recommendations, and the quantity was measured using the Quant-iT^TM^ Picogreen assay kit (Invitrogen, Carlsbad, CA) as directed.

### Genome sequencing and assembly

The draft genome of *C. indolis* was generated at the DOE Joint genome Institute (JGI) using a hybrid of the Illumina and Pacific Biosciences (PacBio) technologies. An Illumina std shotgun library and long insert mate pair library was constructed and sequenced using the Illumina HiSeq 2000 platform [33]. 16,165,490 reads totaling 2,424.8 Mb were generated from the std shotgun and 26,787,478 reads totaling 2,437.7 Mb were generated from the long insert mate pair library. A Pacbio SMRTbellTM library was constructed and sequenced on the PacBio RS platform. 99,448 raw PacBio reads yielded 118,743 adapter trimmed and quality filtered subreads totaling 330.2 Mb. All general aspects of library construction and sequencing performed at the JGI can be found at http://www.jgi.doe.gov. All raw Illumina sequence data was passed through DUK, a filtering program developed at JGI, which removes known Illumina sequencing and library preparation artifacts [34]. Filtered Illumina and PacBio reads were assembled using AllpathsLG (PrepareAllpathsInputs: PHRED 64=1 PLOIDY=1 FRAG COVERAGE=50 JUMP COVERAGE=25; RunAllpath- sLG: THREADS=8 RUN=std pairs TARGETS=standard VAPI WARN ONLY=True OVERWRITE=True) [35]. The final draft assembly contained 1 contig in 1 scaffold. The total size of the genome is 6.4 Mb. The final assembly is based on 2,424.6 Mb of Illumina Std PE, 2,437.6 Mb of Illumina CLIP PE and 330.2 Mb of PacBio post filtered data, which provides an average 759.7× Illumina coverage and 51.6× PacBio coverage of the genome, respectively.

### Genome annotation

Genes were identified using Prodigal [36], followed by a round of manual curation using GenePRIMP [9] for finished genomes and Draft genomes in fewer than 10 scaffolds. The predicted CDSs were translated and used to search the National Center for Biotechnology Information (NCBI) nonredundant database, UniProt, TIGRFam, Pfam, KEGG, COG, and InterPro databases. The tRNAScanSE tool [37] was used to find tRNA genes, whereas ribosomal RNA genes were found by searches against models of the ribosomal RNA genes built from SILVA [38]. Other non–coding RNAs such as the RNA components of the protein secretion complex and the RNase P were identified by searching the genome for the corresponding Rfam profiles using INFERNAL [39]. Additional gene prediction analysis and manual functional annotation was performed within the Integrated Microbial Genomes (IMG) platform [40] developed by the Joint Genome Institute, Walnut Creek, CA, USA [41]. Information in the tables below reflects the gene information in the JGI annotation on the IMG website [40].

## Genome properties

The genome of *C. indolis* DSM 755 consists of a 6,383,701 bp circular chromosome with GC content of 44.93% (Table 3). Of the 5,903 genes predicted, 5,802 were protein-coding genes, and 101 RNAs; 170 pseudogenes were also identified. 81.21% of genes were assigned with a putative function with the remaining annotated as hypothetical proteins. The genome summary and distribution of genes into COGs functional categories are listed in Tables 3 and 4.

**Table 3:** Nucleotide content and gene count levels of the genome of *C. indolis* DSM 755

Attribute	Value	% of total^a^
Genome size (bp)	6,383,701	
DNA Coding region (bp)	5,688,007	89.10
DNA G+C content (bp)	2,868,247	44.93
Total genes^b^	5,903	100.00
RNA genes	101	1.71
Protein-coding genes	5,802	98.29
Protein-coding with function pred.	4,794	81.21
Genes in paralog clusters	4,527	76.69
Genes assigned to COGs	4,643	78.65
Genes with signal peptides	421	7.13
Genes with transmembrane helices	1,494	25.31
Paralogous groups	4,527	76.69

a) The total is based on either the size of the genome in base pairs or the total number of protein coding genes in the annotated genome.

b) Also includes 170 pseudogenes.

**Table 4:** Number of genes in *C. indolis* DSM 755 associated with the 25 general COG functional categories

**Code**	**Value**	**%age**^a^	**Description**
J	184	3.57	Translation
A	0	0	RNA processing and modification
K	531	10.30	Transcription
L	191	3.71	Replication, recombination and repair
B	1	0.02	Chromatin structure and dynamics
D	28	0.54	Cell cycle control, mitosis and meiosis
Y	0	0	Nuclear structure
V	107	2.08	Defense mechanisms
T	335	6.50	Signal transduction mechanisms
M	235	4.56	Cell wall/membrane biogenesis
N	70	1.36	Cell motility
Z	0	0	Cytoskeleton
W	0	0	Extracellular structures
U	41	0.80	Intracellular trafficking and secretion
O	124	2.41	Posttranslational modification, protein turnover, chaperones
C	261	5.06	Energy production and conversion
G	910	17.65	Carbohydrate transport and metabolism
E	493	9.56	Amino acid transport and metabolism
F	110	2.13	Nucleotide transport and metabolism
H	153	2.97	Coenzyme transport and metabolism
I	77	1.49	Lipid transport and metabolism
P	325	6.30	Inorganic ion transport and metabolism
Q	70	1.36	Secondary metabolites biosynthesis, transport and catabolism
R	590	11.45	General function prediction only
S	319	6.19	Function unknown
-	1260	21.35	Not in COGs

a) The total is based on the total number of protein coding genes in the annotated genome.

The genomes of *C. indolis* and its near relatives (*C. saccharolyticum, C. hathewayi, and C. phytofermentans*) have similar numbers of genes in each of the 25 broad COG categories (not shown), however differences exist in the type and distribution of genes in specific functional groups (Table 5), particularly those related to COG categories (G) Carbohydrate transport and metabolism, (C) Energy production and conversion, and (Q) Secondary metabolites biosynthesis, transport and catabolism.

**Table 5:** Number of genes in each of the 25 general COG functional categories^a^ found in *C. indolis* DSM 755^T^ but not in closely related species

**Code**	**Value**	**Description**
J	4	Translation
A	0	RNA processing and modification
K	5	Transcription
L	9	Replication, recombination and repair
B	1	Chromatin structure and dynamics
D	0	Cell cycle control, mitosis and meiosis
Y	0	Nuclear structure
V	1	Defense mechanisms
T	2	Signal transduction mechanisms
M	8	Cell wall/membrane biogenesis
N	2	Cell motility
Z	0	Cytoskeleton
W	0	Extracellular structures
U	1	Intracellular trafficking and secretion
O	10	Posttranslational modification, protein turnover, chaperones
C	28	Energy production and conversion
G	6	Carbohydrate transport and metabolism
E	8	Amino acid transport and metabolism
F	1	Nucleotide transport and metabolism
H	11	Coenzyme transport and metabolism
I	2	Lipid transport and metabolism
P	11	Inorganic ion transport and metabolism
Q	10	Secondary metabolites biosynthesis, transport and catabolism
R	18	General function prediction only
S	21	Function unknown

a) Number of genes from a set of 158 genes not found in near relatives (*C. saccharolyticum, C. phytofermentans, C. hathewayi*) associated with the 25 general COG functional categories.

### Carbohydrate transport and metabolism

**Figure 2:**
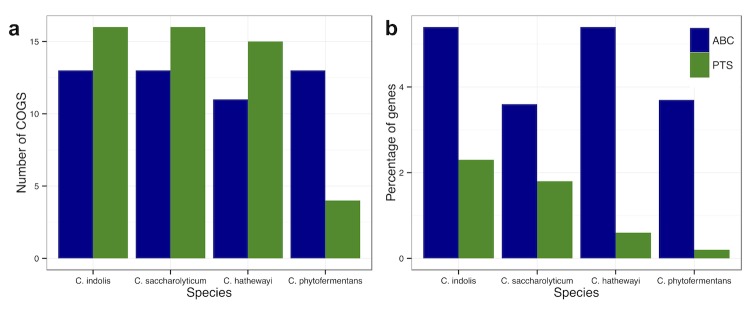
Distribution of ABC and PTS transporters in the genomes of *C. indolis* and related genomes determined from Integrated Microbial Genome (IMG) annotation [40] viewed based on (a) Total umber of COGS, and (b) Percentage of genes in the genome.

Plant biomass is a complex composite of fibrils and sheets of cellulose, hemicellulose, waxes, pectin, proteins, and lignin. Bacteria from soil and the gut generally possess a variety of genes to degrade and transport the diversity of substrates encountered in these plant-rich environments. The genome of *C. indolis* includes 910 genes (17.65% of total protein coding genes) in this COG group including glycoside hydrolases with the potential to degrade complex carbohydrates including starch, cellulose, and chitin (Table 6), as well as an abundance of carbohydrate transporters (Figure 2).

**Table 6:** Selected carbohydrate active genes in the *C. indolis* DSM 755^T^ genome

**Gene count**	**Product name**^a^	**Database ID**_b_
19	Beta-glucosidase (GH-1)	EC:3.2.1.86
8	Beta-galactosidase/ beta-glucuronidase (GH-2)	EC:3.2.1.23 EC:3.2.1.25 EC:3.2.1.31
7	Beta-glucosidase/ related glucosidases (GH-3)	EC:3.2.1.21 EC:3.2.1.52
14	Alpha-galactosidases/ 6-phospho-beta-glucosidases (GH-4)	EC:3.2.1.86 EC:3.2.1.122 EC:3.2.1.22
2	Cellulase, endogluconase (GH-5)	EC:3.2.1.4
14	Alpha-amylase	EC:3.2.1.10 EC:3.2.1.20 EC:2.4.1.7 EC:3.2.1.70
8	Beta-xylosidase (GH 39)	EC:3.2.1.37
2	Chitinase (GH 18)	EC:3.2.1.14

a) GH designations given from the CAZy database [42]. b) Enzyme Commission (EC) numbers assigned by the Integrated Microbial Genome (IMG) database [41].

Almost 8% of the protein-coding genes in the genome of C. indolis were found to be associated with carbohydrate transport, represented by two main strategies. ABC (ATP binding cassette) transporters tend to carry oligosaccharides, and have less affinity for hexoses [43,44], while PTS (phosphotransferase system) transporters carry many different mono- and disaccharides, especially hexoses [45]. PTS systems provide a means of regulation via catabolite repression [46], and are thought to enable bacteria living in carbohydrate-limited environments to more efficiently utilize and compete for substrates [46]. Both *C. indolis* and its near relatives are more highly enriched in ABC than PTS transporters (Fig 2), however nearly a third of *C. indolis* and *C. saccharolyticum* transporters are PTS genes, suggesting a preference for hexoses, as well as an adaptation to more marginal environments. *C. indolis* also possesses ten genes associated with all three components of the TRAP-type C4-dicarboxylate transport system, which transports C4-dicarboxylates such as formate, succinate, and malate [47], as well as six putative malate dehydrogenases and two putative succinate dehydrogenases suggesting that *C. indolis* may have the potential to utilize both of these short chain fatty acids.

### Energy production and conversion

The genome of *C. indolis* contains 261 genes in COG category (C) Energy production and conversion, 28 of which are not found in the near relatives analyzed, including genes for citrate utilization (Table 7) and nitrogen fixation (Table 8).

**Table 7:** Selection of *C. indolis* DSM 755 genes related to citrate utilization.

**Locus Tag**	**Putative Gene Product**_a_	**Gene ID**_a_
K401DRAFT_2892	holo-ACP synthase (CitX)	EC:2.7.7.61
K401DRAFT_2893	citrate lyase acyl carrier (CitD)	EC:4.1.3.6
K401DRAFT_2894	citrate lyase beta subunit (CitE)	EC:4.1.3.6 EC:2.8.3.10
K401DRAFT_2895	citrate lyase alpha subunit (CitF)	EC:4.1.3.6 EC:2.8.3.10
K401DRAFT_2896	triphosphoribosyl-dephospho-CoA synthase (CitG)	EC:2.7.8.25
K401DRAFT_2897	citrate (pro3S)-lyase ligase (CitC)	EC:6.2.1.22
K401DRAFT_2898	response regulator, CheY-like receiver domain, winged helix DNA binding domain	-
K401DRAFT_2899	signal transduction histidine kinase	-
K401DRAFT_2900	citrate transporter, CITMHS family	KO:K03303 TC.LCTP

Gene products and Enzyme Commission (EC) numbers assigned by the Integrated Microbial Genome (IMG) database [41].

**Table 8:** Selection of *C. indolis* DSM 755 genes related to nitrogen fixation.

**Locus Tag**	**Putative Gene Product**	**Gene ID**
K401DRAFT_0533	nitrogenase Mo-Fe protein, α and β chains	pfam00148
K401DRAFT_0534	nitrogenase Mo-Fe protein, α and β chains	pfam00148
K401DRAFT_0535	nitrogenase subunit (ATPase) (nifH)	pfam00142
K401DRAFT_0884	nitrogenase Mo-Fe protein, α and β chains	pfam00148
K401DRAFT_0885	nitrogenase Mo-Fe protein, α and β chains	pfam00148
K401DRAFT_0886	nitrogenase subunit (ATPase) (nifH)	pfam00142
K401DRAFT_3349	nitrogenase Mo-Fe protein, α and β chains	pfam00148
K401DRAFT_3350	nitrogenase Mo-Fe protein, α and β chains	pfam00148
K401DRAFT_3351	nitrogenase subunit (ATPase) (nifH)	pfam00142
K401DRAFT_3874	nitrogenase Mo-Fe protein, α and β chains (nifD)	pfam00148
K401DRAFT_3875	nitrogenase Mo-Fe protein, α and β chains (nifK)	pfam00148
K401DRAFT_3876	nitrogenase Fe protein	pfam00142
K401DRAFT_3878	nitrogenase Mo-Fe protein, α and β chains (nifD)	pfam00148
K401DRAFT_3879	nitrogenase Mo-Fe protein, α and β chains (nifK)	pfam00148
K401DRAFT_3880	dinitrogenase Fe-Mo cofactor, (nifH)	pfam02579
K401DRAFT_3895	nitrogenase Mo-Fe protein, α and β chains (nifD)	pfam00148
K401DRAFT_3896	nitrogenase Mo-Fe protein, α and β chains (nifK)	pfam00148
K401DRAFT_5519	nitrogenase Mo-Fe protein, α and β chains (nifB)	pfam04055
K401DRAFT_5520	nitrogenase Mo-Fe protein, α and β chains (nifE)	pfam00148
K401DRAFT_5521	nitrogenase Mo-Fe protein (nifK)	pfam00148
K401DRAFT_5522	nitrogenase component 1, alpha chain (nifN-like)	pfam00148
K401DRAFT_5525	nitrogenase subunit (ATPase) (nifH)	pfam00142

Nitrogenase genes have a common gene identifier (EC:1.18.6.1), therefore the pfam numbers are given to distinguish between subunits. Gene product names and pfam numbers assigned by the Integrated Microbial Genome (IMG) database [41].

### Citrate utilization

**Figure 3:**
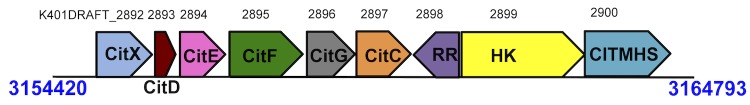
Citrate utilization genes are in a single gene cluster on K401DRAFT_scaffold0000.1.1, including the citrate transporter CitMHS, and a putative two-component system.

Citrate is a metabolic intermediary found in all living cells. In aerobic bacteria, citrate is utilized as part of the tricarboxylic acid (TCA) cycle. In anaerobes, citrate is fermented to acetate, formate, and/or succinate. The first step is the conversion of citrate to acetate and oxaloacetate in a reaction catalyzed by citrate lyase (EC:4.1.3.6) [48]. *C. sphenoides*, a close relative of *C. indolis* that does not yet have a sequenced genome has been shown to utilize citrate [49], but there is conflicting evidence as to whether this phenotype is present in *C. indolis* [28,30]. The genome of *C. indolis* reveals a group of seven citrate genes organized in a cluster similar to operons found in other bacterial species [48,50] (Figure 3) including CitD, CitE, and CitF, the three subunits of the citrate lyase gene [48], CitG and CitX which have been shown to be necessary for citrate lyase function [50], CitMHS, a citrate transporter, and a putative two component system similar to citrate regulatory mechanisms in other bacteria [51].

### Nitrogen Fixation

Nitrogen fixation has been observed in other clostridia [52,53] but has not been demonstrated in the *C. saccharolyticum* species group. It has been suggested that the capacity to fix nitrogen confers a selective advantage to cellulolytic microbes that live in nitrogen limited environments such as many soils [52]. The functional summary suggests that *C. indolis* can fix nitrogen. The *C. indolis* genome reveals 22 nitrogenase related genes in four gene clusters (Table 8), none of which are found in the near relatives analyzed in this study. A minimum set of six genes encoding for structural and biosynthetic components of a functional nitrogenase complex have been hypothesized [54]. Genes needed for the nitrogenase structural component proteins (*nifH*, *nifD*, and *nifK*) are present in *C. indolis*, but one of the three genes required to synthesize the nitrogenase iron-molybdenum cofactor (*nifN*) is not identified. Follow up experiments are needed to determine whether *C. indolis* can fix nitrogen as predicted by the genome analysis.

### Lactate utilization

The genome of *C. indolis* includes both D- and L-lactate dehydrogenases, which convert lactate to pyruvate. Additionally, there is a lactate transporter, suggesting that *C. indolis* is able to utilize exogenous lactate [Table 9].

**Table 9:** Selection of *C. indolis* DSM 755 genes related to lactate utilization.

**Locus Tag**	**Putative Gene Product**	**Gene ID**
K401DRAFT_1877	L-lactate dehydrogenase	EC:1.1.1.27
K401DRAFT_5775	L-lactate dehydrogenase	EC:1.1.1.27
K401DRAFT_3431	L-lactate transporter, LctP family	TC.LCTP
K401DRAFT_3220	D-lactate dehydrogenase	EC:1.1.1.28

Annotations assigned by the Integrated Microbial Genome (IMG) database [41]

### Bacterial microcompartments (BMC)

The *C. indolis* genome contains genes associated with bacterial microcompartment shell proteins. Bacterial microcompartments (BMCs) are proteinaceous organelles involved in the metabolism of ethanolamine, 1,2-propanediol, and possibly other metabolites (Rev in [55-57]). BMCs are often encoded by a single operon or contiguous stretch of DNA. The different metabolic types of BMCs can be distinguished by a key enzyme (e.g., ethanolamine lyase and propanediol dehydratase) related to its metabolic function. While the other associated genes in the operon can vary, they frequently include an alcohol dehydrogenase, an aldehyde dehydrogenase, an aldolase and an oxidoreductase.

**Figure 4:**
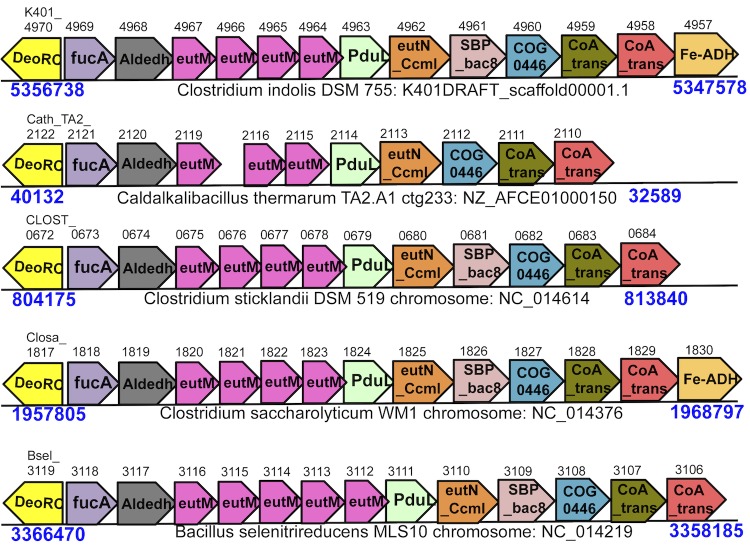
CoAT BMC operon found in *C. indolis, Caldalkalibacillus thermarum, C. stricklandii, C. saccharolyticum,* and *Bacillus selenitrireducens*. Gene details are found in Table 11.

In *C. indolis* there are 2 separate genetic loci that code for BMCs (Table 10 and 11 and Figure 4). One *C. indolis* locus (Table 10) contains a gene (K401DRAFT_2189) with sequence similarity to a B_12_-independent propanediol dehydratase found in *Roseburia inulinivorans* and *Clostridium phytofermentans* [58,59] (both members of the *Lachnospiraceae*). This enzyme has been shown to be involved in the metabolism of fucose and rhamnose [58,59] and was subsequently categorized as the glycyl radical prosthetic group-based (grp) BMC [60]. The glycyl radical family of enzymes was recently expanded to include a choline trimethylamine lyase activity that is part of a microcompartment loci in *Desulfovibrio desulfuricans* [61]. The corresponding C. indolis** enzymes (K401DRAFT_2189 and K401DRAFT_2190) are more similar to the *D. desulfuricans* protein, but there are differences in the gene content of the microcompartment loci. Further work is needed to determine the physiological role of this microcompartment.

**Table 10:** grp-BMC genes found in the *C. indolis* genome.

**Locus Tag**	**Product Name**	**Gene ID/ Protein Information**
K401DRAFT_2181	Predicted transcriptional regulator	COG0789
K401DRAFT_2182	Predicted membrane protein	COG2510
K401DRAFT_2183	Carbon dioxide concentrating mechanism/carboxysome shell protein	pfam00936
K401DRAFT_2184	Predicted membrane protein	pfam00936
K401DRAFT_2185	Hypothetical protein	-
K401DRAFT_2186	Carbon dioxide concentrating mechanism/carboxysome shell protein	pfam00936
K401DRAFT_2187	Carbon dioxide concentrating mechanism/carboxysome shell protein	pfam00936
K401DRAFT_2188	NAD-dependent aldehyde dehydrogenase	pfam00171
K401DRAFT_2189	Pyruvate formate lyase	pfam02901
K401DRAFT_2190	Pyruvate formate lyase activating enzyme	pfam04055
K401DRAFT_2191	Ethanolamine utilization protein	pfam00936
K401DRAFT_2192	Ethanolamine utilization protein	pfam10662
K401DRAFT_2193	Alcohol dehydrogenase, class IV	pfam00465
K401DRAFT_2194	Ethanolamine utilization cobalamin adenosyltransferase	COG4892
K401DRAFT_2195	Ethanolamine utilization protein, possible chaperonin	COG4820
K401DRAFT_2196	Carbon dioxide concentrating mechanism/carboxysome shell protein	pfam00936
K401DRAFT_2197	Carbon dioxide concentrating mechanism/carboxysome shell protein	pfam03319
K401DRAFT_2198	Ethanolamine utilization protein	pfam06249
K401DRAFT_2199	Carbon dioxide concentrating mechanism/carboxysome shell protein	pfam00936
K401DRAFT_2200	NAD-dependent aldehyde dehydrogenase	pfam00171
K401DRAFT_2201	Propanediol utilization protein	pfam06130
K401DRAFT_2202	Carbon dioxide concentrating mechanism/carboxysome shell protein	pfam00936

Annotations assigned by the Integrated Microbial Genome (IMG) database [41].

**Table 11:** CoAT BMC genes found in the *C. indolis* genome.

**Locus Tag**	**Product Name**	**Gene ID/ Protein Information**
K401DRAFT_4970	DeoRC transcriptional regulator	pfam00455
K401DRAFT_4969	fucA, L-fuculose-phosphate aldolase	EC:4.1.2.17
K401DRAFT_4968	pduP, propionaldehyde dehydrogenase	pfam00171
K401DRAFT_4967	eutM, ethanolamine utilization protein	pfam00936
K401DRAFT_4966	Carbon dioxide concentrating mechanism/carboxysome shell protein	pfam00936
K401DRAFT_4965	Carbon dioxide concentrating mechanism/carboxysome shell protein	pfam00936
K401DRAFT_4964	Carbon dioxide concentrating mechanism/carboxysome shell protein	pfam00936
K401DRAFT_4963	Pdul, propanediol utilization protein	pfam06130
K401DRAFT_4962	eutN_CcmL	pfam03319
K401DRAFT_4961	SBP_bac_8, ABC-type sugar transporter	pfam13416
K401DRAFT_4960	Uncharacterized NAD(FAD)-dependent dehydrogenase	COG0446
K401DRAFT_4959	CoA-transferase	pfam01144
K401DRAFT_4958	CoA-transferase	pfam01144
K401DRAFT_4957	Fe-ADH, Alcohol dehydrogenase	pfam00465

Annotations assigned by the Integrated Microbial Genome (IMG) database [41]

The second *C. indolis* BMC loci (Table 11 and Figure 4) is even more enigmatic. This loci contains the shell proteins, alcohol dehydrogenase, aldehyde dehydrogenase, aldolase and oxidoreductase commonly found in microcompartments, but it lacks a known key enzyme. Homologs of this operon were found in four other bacterial species (Figure 4). They are all missing a known key enzyme and contain 2 genes annotated as CoA-transferase. We propose that the *C. indolis* genome and these other bacteria contain a novel type of microcompartment, designated the CoAT BMC. It is not clear that the function of the 2 annotated CoA-transferase genes are as predicted and further research is needed to demonstrate the physiological role of this BMC.

### Secondary metabolites biosynthesis, transport and catabolism

Protocatechuate and other aromatics are intermediaries in the degradation of lignin in plant rich environments [62]. The genome of *C. indolis* contains two protocatechuate dioxygenases and an aromatic hydrolase, revealing the potential for utilizing aromatic compounds (Table 12).

**Table 12:** Selection of *C. indolis* DSM 755^T^ genes related to degradation of aromatics.

**Locus Tag**	**Putative Gene Product**	**Gene ID**
K401DRAFT_3571	Protocatechuate 3,4-dioxygenase beta subunit	EC:1.13.11.3
K401DRAFT_3568	Protocatechuate 3,4-dioxygenase beta subunit	EC:1.13.11.3
K401DRAFT_3412	Aromatic ring hydroxylase	EC:5.3.3.3 EC:4.2.1.120

Annotations assigned by the Integrated Microbial Genome (IMG) database [41]

## Conclusion

The genomic sequence of *C. indolis* reported here reveals the metabolic potential of this organism to utilize a wide assortment of fermentable carbohydrates and intermediates including citrate, lactate, malate, succinate, and aromatics, and points to potential ecological roles in nitrogen fixation and ethanolamine utilization. Further culture-based characterization is necessary to confirm the metabolic activity suggested by this genomic analysis, and to expand the description of *C. indolis*.
